# Evaluation of Lymphatic Filariasis and Onchocerciasis in Three Senegalese Districts Treated for Onchocerciasis with Ivermectin

**DOI:** 10.1371/journal.pntd.0005198

**Published:** 2016-12-07

**Authors:** Nana O. Wilson, Alioune Badara Ly, Vitaliano A. Cama, Paul T. Cantey, Daniel Cohn, Lamine Diawara, Abdel Direny, Mawo Fall, Karla R. Feeser, LeAnne M. Fox, Achille Kabore, Amadou F. Seck, Ngayo Sy, Daouda Ndiaye, Christine Dubray

**Affiliations:** 1 Division of Parasitic Diseases and Malaria, Center for Global Health, Centers for Disease Control and Prevention (CDC), Atlanta, Georgia, United States of America; 2 Ministère de la Santé et de l’Action Sociale, Dakar, Senegal; 3 RTI International, Washington, DC, United States of America; 4 World Health Organization, Regional Office for Africa, Senegal; 5 African Programme for Onchocerciasis Control Representative, Ougadougou, Burkina Faso; 6 IMA World Health, Port-au-Prince, Haiti; 7 RTI/ENVISION, Dakar, Senegal; 8 Service de Lutte Antiparasitaire, Ministère de la Santé et de l’Action Sociale, Thies, Senegal; 9 University Cheikh Anta Diop, Dakar, Senegal; University of South Florida, UNITED STATES

## Abstract

In Africa, onchocerciasis and lymphatic filariasis (LF) are co-endemic in many areas. Current efforts to eliminate both diseases are through ivermectin-based mass drug administration (MDA). Years of ivermectin distribution for onchocerciasis may have interrupted LF transmission in certain areas. The Kédougou region, Senegal, is co-endemic for LF and onchocerciasis. Though MDA for onchocerciasis started in 1988, in 2014 albendazole had not yet been added for LF. The objective of this study was to assess in an integrated manner the LF and onchocerciasis status in the three districts of the Kédougou region after ≥10 years of ivermectin-based MDA. The study employed an African Programme for Onchocerciasis Control (APOC) onchocerciasis-related methodology. In the three districts, 14 villages close to three rivers that have *Simulium damnosum* breeding sites were surveyed. Convenience sampling of residents ≥5 years old was performed. Assessment for LF antigenemia by immunochromatographic testing (ICT) was added to skin snip microscopy for onchocerciasis. Participants were also tested for antibodies against Wb123 (LF) and Ov16 (onchocerciasis) antigens. In two districts, no participants were ICT or skin snip positive. In the third district, 3.5% were ICT positive and 0.7% were skin snip positive. In all the three districts, Wb123 prevalence was 0.6%. Overall, Ov16 prevalence was 6.9%. Ov16 prevalence among children 5–9 years old in the study was 2.5%. LF antigenemia prevalence was still above treatment threshold in one district despite ≥10 years of ivermectin-based MDA. The presence of Ov16 positive children suggested recent transmission of *Onchocerca volvulus*. This study showed the feasibility of integrated evaluation of onchocerciasis and LF but development of integrated robust methods for assessing transmission of both LF and onchocerciasis are needed to determine where MDA can be stopped safely in co-endemic areas.

## Author Summary

Onchocerciasis and lymphatic filariasis (LF) are neglected tropical diseases caused by infection with filarial parasites, *Onchocerca volvulus* and *Wuchereria bancrofti* or *Brugia* species respectively. Efforts to eliminate both diseases are through ivermectin-based mass drug administration (MDA) for onchocerciasis and MDA with albendazole and ivermectin or diethylcarbamazine for LF. Both diseases overlap significantly in Africa and years of ivermectin-based MDA for onchocerciasis in co-endemic areas may have decreased or interrupted *W*. *bancrofti* transmission. Senegal is co-endemic for both diseases. As of 2014, 37 LF endemic districts had not received specific treatment for LF; however, eight of these districts had been receiving ivermectin-based MDA for onchocerciasis for more than 10 years. Here we estimated, using an APOC onchocerciasis-related methodology, the status of LF and onchocerciasis in three districts of Senegal after more than 10 years of ivermectin-based MDA for onchocerciasis. We demonstrated the feasibility of integrated evaluation of LF and onchocerciasis. The results showed that *W*. *bancrofti* antigenemia prevalence in one district was ≥1%. In the districts where we did not detect LF antigenemia among the participants a more robust evaluation like a transmission assessment survey among children is still needed to determine if the infection rate for *W*. *bancrofti* is below the transmission threshold and MDAs for LF are not needed. Despite the small sample of children included in this study, the onchocerciasis antibody prevalence among them was above the threshold recommended to stop MDA. This study provides another example of the complexities that encompass stop MDA decision-making in onchocerciasis and LF co-endemic areas.

## Introduction

Lymphatic filariasis (LF) is an infection caused by the parasitic nematodes *Wuchereria bancrofti*, *Brugia malayi*, and *Brugia timori* and transmitted by mosquitoes. LF is endemic in 73 countries with an estimated 1.1 billion people at risk who required mass drug administration (MDA) in 2014 [1]. LF is targeted for global elimination as a public health problem by 2020 (World Health Assembly resolution 50.29). In Africa, the elimination strategy is through annual MDA with albendazole and ivermectin or diethylcarbamazine.

Onchocerciasis is an infection caused by filarial parasite *Onchocerca volvulus* and transmitted by black flies belonging to the genus *Simulium*. At least 169 million people are estimated to be at risk of infection in 31 countries in Africa [2]. The Onchocerciasis Control Programme (OCP) was launched in 1975 in 11 West African countries to control onchocerciasis mostly through vector control [3]. The program ended in 2002 and was replaced by the African Programme for Onchocerciasis Control (APOC) to control onchocerciasis through annual or six-monthly ivermectin-based MDA [4]. APOC goals transitioned from control to elimination of onchocerciasis after a study published in 2009 that took place in Mali and Senegal provided the first evidence that the elimination of onchocerciasis was feasible in Africa with ivermectin distribution alone [5].

In Africa, there is significant overlap between LF and onchocerciasis [6]. However, few studies have attempted to assess the impact of onchocerciasis interventions on LF in co-endemic areas in the absence of albendazole MDA. In a study conducted in Burkina Faso to look at LF prevalence [7], six communities that received ivermectin MDA for six years for onchocerciasis had a filarial circulating antigen (immunochromatographic test, ICT) positivity rate of 37.8% compared to 48.5% for five communities that had not received ivermectin MDA. In addition, the LF microfilaremia rates were 6.9% and 15.9% in ivermectin treated and untreated communities respectively. In another study in Burkina Faso [8], eight communities that had received ivermectin for 14 years were compared to eight that had not received ivermectin. Night blood film examination found no evidence of LF infection in the eight treated villages and 2.9% positives in the untreated villages. In a study in Nigeria [9], ICT antigenemia rates between five treated villages and one untreated village differed significantly, 41% versus 58%. The same study compared anopheline mosquito infection rates between six communities receiving a total of two or five rounds of ivermectin with five communities that had not received ivermectin and found no difference in infection rates. Finally, in a study in north-west Ethiopia, LF transmission was not interrupted in three villages treated during seven years with annual mass treatment with ivermectin monotherapy for onchocerciasis. The baseline mapping results showed antigenemia prevalence ranging from 23 to 56% in these villages. After seven years of ivermectin treatment the mean microfilaremia prevalence in the three villages was still 4.7% [10]. These studies suggest that annual ivermectin may reduce prevalence of infection but may not be sufficient to interrupt transmission.

**Fig 1:**
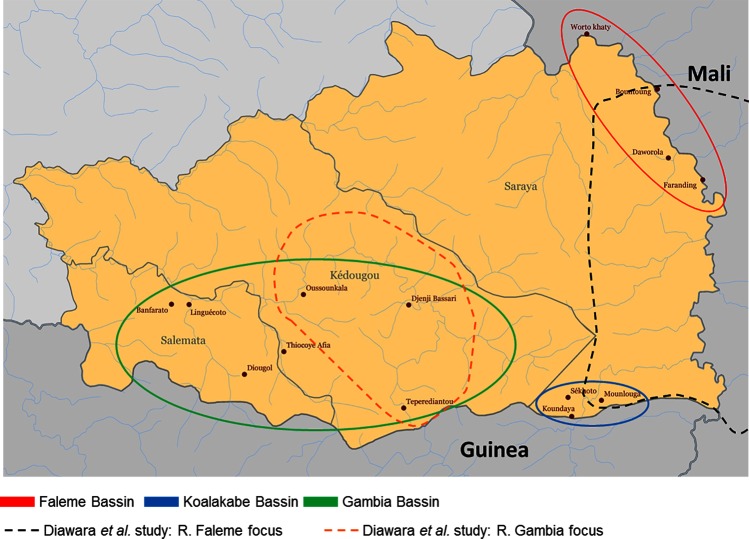
Villages included in the study by district and river and in comparison with [5] Diawara et al., 2009 study sites, Kédougou region, Senegal.

Senegal’s goal is to eliminate onchocerciasis, where possible, via preventive mass ivermectin treatment. Eight of the 76 health districts are endemic for onchocerciasis (three districts in Kédougou Region, one district in Kolda Region, and four districts in Tambacounda Region). Onchocerciasis control based on ivermectin treatment started in 1988. Initially the National Onchocerciasis Control Program (PNLO) provided treatment through community-directed treatment with ivermectin (CDTI) in endemic foci (hyper, meso, and hypo-endemic). In 2009, a study showed that *O*. *volvulus* transmission has been interrupted in some foci after 15–17 years of ivermectin MDA [5, 11]. No recrudescence of transmission was detected five years after the last round of ivermectin treatment, and infectivity in flies continued to decrease [11]. The areas where these studies were conducted are shown in Fig 1. Nonetheless, in 2012 in Tambacounda, and in 2013 in Kédougou and Kolda the PNLO decided to resume onchocerciasis treatment and the Ministry of Health shifted to a MDA model, integrating the treatment for onchocerciasis with MDA for LF, which is also endemic.

Senegal’s goal is to eliminate LF as a public health problem by 2020. LF has been fully mapped in Senegal: 14 districts were mapped in 2003, 6 in 2007, 27 in 2010, and the final 6 districts were mapped in 2012. A total of 50 districts are LF-endemic across 12 of the country’s 14 regions. In 2014 when this study took place, 37 of the LF endemic districts were not receiving treatment, of which eight had been receiving ivermectin MDA for onchocerciasis for more than 10 years. In 2015, all 50 LF-endemic districts were treated, 37 of them for the first time.

In early 2014, the PNLO planned to perform onchocerciasis epidemiological and entomological evaluations in the Kédougou Region to assess if *O*. *volvulus* transmission had been interrupted and ivermectin-based MDA could be stopped. The Ministry of Health was interested to know the status of LF in these same districts since the LF mapping in 2003 showed that LF prevalence was above the treatment threshold and albendazole had never been included in MDAs. Therefore, the objective of this study was to assess the status of LF and onchocerciasis in the three districts of the Kédougou Region in Senegal after more than 10 years of ivermectin-based MDAs for onchocerciasis in an integrated manner using the APOC onchocerciasis-related methodology to confirm that the breakpoint has been reached and that treatment can be safely stopped using prevalence of microfiladermia and entomological evaluation (separate from the study). In addition, we compared the antibody response to the *W*. *bancrofti* antigen Wb123 and *O*. *volvulus* antigen Ov16 to diagnostic methods used by LF and onchocerciasis programs.

## Methods

### Ethics statement

This study was conducted by the National Neglected Tropical Diseases Program of the Ministry of Health, Senegal. Ethical approval for the study was obtained from the National Ethical Committee for Health Research (NERS) of the Ministry of Health of Senegal and the Institutional Review Board at the Centers for Disease Control and Prevention (CDC), Atlanta, USA. Written informed consent was obtained from participants before samples were collected. Children under the age of 18 years were recruited into the study after permission was obtained from their parent or legal guardian. Assent was obtained from children 10–17 years old.

### Study sites

The study took place in the three districts (Kédougou, Saraya, and Salemata) of the Kédougou region. The total populations of the Kédougou, Saraya, and Salemata districts were 76,242, 54,659, and 21,233 (census 2013), respectively. In these districts, the PNLO selected 14 villages located in the Gambia River focus, the Koalakabe River focus, and the Faleme River focus. The 14 villages were from high-risk areas near the rivers and breeding sites for the vector *Simulium damnosum*. The map presented in Fig 1 shows the villages selected and their locations compared to rivers and districts (solid line). Some but not all the villages included in this study were part of the areas covered by the Diawara *et al*. study in the Gambia and Faleme foci (dash lines) [5]. During the 2003 mapping for LF, the prevalence of adult filarial antigenemia by ICT was ≥1% in the three villages selected for the mapping (19% in one village and 5% in each of the other two villages). These three villages were not part of the villages selected by the PNLO for this study. When this study was implemented in January 2014, MDA with albendazole for LF had not been started in the Kédougou region. In the villages selected for this study, ivermectin-based MDA for onchocerciasis started in 1988–1989 [12]. Ivermectin treatment was given in April or May just before the rainy season in order to optimize the impact of treatment on the transmission [5, 11]. In the River Gambia focus, treatment was given at six-monthly intervals from 1990 onwards [5]. Table 1 shows the number of years of MDA by village from 1988 to 2012 and the range or treatment coverage (1988–2007) reported by the Ministry of Health in Senegal. Therefore the districts included in this study had the following characteristics: (i) they had been treated with ivermectin for onchocerciasis for >10 years; (ii) they were likely or known to be co-endemic for LF and onchocerciasis; (iii) preventive chemotherapy for LF with albendazole had not yet been started or implemented. It is important to note that this study took place at least 11 months after the last ivermectin treatment.

**Table 1:** Number of ivermectin mass drug administration round for onchocerciasis by river and village and reported coverage

River	Village	Year of 1^st^ MDA reported	Last MDA reported	Number of years MDA reported before survey	Range of reported coverage
**Gambia**	Banfarato	1989	2010	15	61–87%
	Linguécoto	1988	2009	20	61–90%
	Diougol	1989	2010	16	45–88%
	Thiocoye Afia	1989	2010	14	62–87%
	Oussounkala	1988	2011	22	66–93%
	Djenji Bassiri	1988	2007	20	59–86%
	Teperediantou	1989	2009	20	70–89%
**Koalakabe**	Koundaya	1989	2010	11	68–92%
	Sékhoto	1990	2007	16	71–97%
	Mounlouga	1989	2006	15	41–92%
**Faleme**	Worto Khaty	2001	2011	5	71–84%
	Bountoung	1998	2000	8	15–90%
	Daworola	1990	2012	11	64–100%
	Faranding	1989	2007	12	61–100%

MDA–mass drug administration

### Study design

LF evaluation components were added to the standard onchocerciasis epidemiological evaluation used by APOC to assess the impact of ivermectin-based MDAs on *O*. *volvulus* transmission. The sampling criteria were defined by the Senegal National Onchocerciasis Control Program following the recommendation outlined in the APOC Conceptual and Operational Framework of Onchocerciasis Elimination with Ivermectin Treatment [13]. The evaluation was performed at least 11 months after the last ivermectin treatment. A community census was performed to define the total population of the selected villages, and any resident age five years and older willing to participate was included in the study.

### Sampling and diagnostic tests

In each selected village, all eligible people who agreed to participate in the study (convenience sampling) were examined for onchocerciasis and LF. The study used the established skin snip examination method to determine *O*. *volvulus* infection. Briefly, two skin snips were taken from the iliac crests with a sterile 2 mm Holth corneoscleral punch biopsy tool and microscopically examined for the presence of *O*. *volvulus* microfilariae after incubation for 30 minutes in distilled water and a further 24 hours in saline for negative skin snips. In addition to the skin snip examination, a total of 160μl of capillary blood was collected from each participant, 100μl were used for the ICT test for filarial antigen, and 60μl were used for dried blood spots (DBS) on filter paper. The DBS were transported to the CDC, Atlanta, USA for multiplex bead assay (Luminex) for antibody reactivity to Ov16 and Wb123 antigens. Information such as location, demographics, and migration history for each person was collected on a personal digital assistant (PDA) (Hewlett Packard iPAQ 211). Each participant was assigned a unique identifier number that was printed on a barcode label. Barcode labels were affixed to the ICT cards and to the filter paper of each participant. External global positioning system (GPS) cards (GlobalSat BC-337) were attached to the PDA to track GPS coordinates of each village.

### Serologic testing

The Ov16 and Wb123 multiplex bead assay uses recombinant antigens of *O*. *volvulus* and *W*. *bancrofti* respectively to measure the prevalence of IgG4 antibodies. A total of 40μl of serum was eluted from DBS for use in the multiplex bead assay. The assay measured fluorescence intensity from antigen-coupled beads. The fluorescence was reported as median fluorescent intensity minus background (MFI-bg) using Bio-Plex 200 system. The cut-off limit of each antigen was determined by receiver operating characteristics (ROC) analysis of MFI-bg with a panel of 91 defined specimens.

### Field activities

To ensure standardization of enrollment and data collection, a three-day training session was conducted for field staff. The team met with community leaders and the village population to explain the nature of the study and its significance, and respond to questions before the study was conducted. Six stations were organized in the middle of each selected village for the informed consent, the census, the questionnaire, the physical examination, the capillary blood collection for ICT and DBS, and the skin snip collection.

### Data analysis

Frequencies and 95% confidence interval (CI) for skin snip, LF antigenemia, Wb123 and Ov16 positivity were determined using STATA 13 (StataCorp LP, College Station TX). The 95% CI of the prevalence or frequencies was calculated by using binomial confidence interval exact test calculator. Logistic regression analysis was used to determine the risk factors associated with ICT, Wb123, and Ov16 positivity. Statistical significance was determined at the 5% level.

## Results

### Participant characteristics

In the 14 villages, 2328 individuals were eligible and 51.2% (1192/2328) consented to participate in the study. Among the participants who consented, 96.8% (1154/1192) completed the questionnaire. The median age of the participants was 15 years (range, 5–100 years) and 52.3% were male (604/1154). A total of 286 (24.8%) participants were <10 years old. The median number of years participants had lived in the villages was 13; 7.1% (82/1154) reported living outside the village for more than six months in the last 10 years, and 63.4% (732/1154) reported taking ivermectin in the past. The occupations reported were student (44.4%), farmer (38.6%), housewife (15.4%), gold miner (13%), other (2.9%), teacher (1.1%), and fisherman (0.6%).

### LF infection by district

Of the individuals who completed the questionnaire, 99% (1145/1154) provided a capillary blood specimen for ICT test and DBS on filter paper. The overall LF antigenemia prevalence in the three districts was 0.5% (95% CI = 0.2–1.2%) (Table 2). All antigen positive participants were from Salemata district (n = 6, 3.5% prevalence, 95% CI = 1.3–7.5%). The age range of the antigenemia positive participants was 25–79 years and 50% of them were male. In the three districts, the seroprevalence for Wb123 antibodies was 0.6% (n = 7, 95% CI = 0.2–1.3%) (Table 2). None of the antigenemia positive participants were Wb123 positive. The age range of the participants with positive antibody results was 7–69 years and 86% were male. There were no significant associations between demographic characteristics and LF antigenemia or Wb123 prevalence (Table 3).

**Table 2:** Prevalence of lymphatic filariasis positive results by district and village, Kédougou Region, using two diagnostic tests

District	Village	ICT*		Wb123**	
		n/N (%)	95% CI	n/N (%)	95% CI
**Kédougou**	Djenji Bassiri	0/35 (0)	0.0–14.9	0/35 (0)	0.0–14.9
	Oussounkala	0/173 (0)	0.0–3.2	1/173 (0.6)	0.0–3.2
	Teperediantou	0/143 (0)	0.0–3.8	0/143 (0)	0.0–3.8
	Thiocoye Afia	0/31 (0)	0.0–16.7	0/31 (0)	0.0–16.7
	**Total**	**0/382 (0)**	**0.0–1.4**	**1/382 (0.3)**	**0.0–1.4**
**Saraya**	Bountoung	0/78 (0)	0.0–6.9	1/78 (1.3)	0.0–6.9
	Daworola	0/90 (0)	0.0–6.0	0/90 (0)	0.0–6.0
	Faranding	0/93 (0)	0.0–5.8	0/93 (0)	0.0–5.8
	Koundaya	0/85 (0)	0.0–6.4	3/85 (3.5)	0.7–10.0
	Mounlouga	0/58 (0)	0.0–9.2	0/58 (0)	0.0–9.2
	Sékhoto	0/84 (0)	0.0–6.5	0/84 (0)	0.0–6.5
	Worto Khaty	0/90 (0)	0.0–6.0	0/90 (0)	0.0–6.0
	**Total**	**0/578 (0)**	**0.0–0.9**	**4/578 (0.7)**	**0.2–1.8**
**Salemata**	Banfarato	1/51 (2)	0.0–10.5	0/51 (0)	0.0–10.5
	Diougol	3/47 (6.4)	1.3–17.5	0/47 (0)	0.0–11.3
	Linguécoto	2/73 (2.7)	0.3–9.6	2/73 (2.7)	0.3–9.6
	**Total**	**6/171 (3.5)**	**1.3–7.5**	**2/171 (1.2)**	**0.1–4.2**
	**Grand Total**	**6/1131 (0.5)**	**0.2–1.2**	**7/1131 (0.6)**	**0.2–1.3**

* Antigenic immunochromatographic card test

** Antibody response to the *W*. *bancrofti* antigen Wb123

**Table 3:** Prevalence of lymphatic filariasis positive results by age and gender, Kédougou Region, using two diagnostic tests

	ICT*			Wb123**		
	n/N (%)	OR	95% CI	n/N (%)	OR	95% CI
**Age group (years)**						
5–9	0/279 (0)	1		1/279 (0.4)	1	
10–14	0/282 (0)	0.4	0.1–2.0	1/282 (0.4)	1.0	0.1–15.9
≥15	6/570 (1.1)	0.8	0.3–2.4	5/570 (0.9)	2.5	0.3–21.2
**Gender**						
Female	3/559 (0.5)	1		1/559 (0.2)	1	
Male	3/572 (0.5)	0.8	0.2–3.9	6/572 (1.0)	5.1	0.6–43.2

* Antigenic immunochromatographic card test

** Antibody response to the *W*. *bancrofti* antigen Wb123

### Onchocerca volvulus infection by river foci

Of the individuals who completed the questionnaire, 86% (992/1154) consented to skin snip examination. The prevalence of microfiladermia among participants was 0.1% (n = 1, 95% CI = 0.1–0.6%) (Table 4). The single skin snip positive participant was a 79-year-old male from the Gambia River focus and came from the Linguécoto village (Table 4). The community of the Sékhoto village refused skin snip entirely. There were no palpable nodules or skin manifestations of onchocerciasis observed among the participants of the study.

**Table 4:** Prevalence of onchocerciasis positive results by river and village, Kédougou Region

River	Village	Skin Snip		OV-16*	
		n/N (%)	95% CI	n/N (%)	95% CI
**Gambia**	Banfarato	0/35 (0)	0.0–14.9	2/51 (3.9)	0.5–13.5
	Diougol	0/47 (0)	0.0–11.3	0/47 (0)	0.0–11.3
	Linguécoto	1/73 (1.4)	0.0–7.4	0/73 (0)	0.0–7.4
	Djenji Bassiri	0/36 (0)	0.0–14.5	3/35 (8.6)	1.8–23.1
	Oussounkala	0/175 (0)	0.0–3.1	14/173 (8.1)	4.5–13.2
	Teperediantou	0/137 (0)	0.0–4.0	15/143 (10.5)	6.0–16.7
	Thiocoye Afia	0/27 (0)	0.0–19.0	5/31 (16.1)	5.5–33.7
	**Total**	**1/530 (0.2)**	**0.0–1.0**	**39/553 (7.0)**	**5.1–9.5**
**Faleme**	Bountoung	0/74 (0)	0.0–7.3	1/78 (1.3)	0.0–6.9
	Daworola	0/92 (0)	0.0–5.9	4/90 (4.4)	1.2–11.0
	Faranding	0/88 (0)	0.0–6.2	5/93 (5.4)	1.8–12.1
	Worto Khaty	0/75 (0)	0.0–7.2	5/90 (5.6)	1.8–12.5
	**Total**	**0/329 (0)**	**0.0–1.7**	**15/351 (4.3)**	**2.4–6.9**
**Koalakabe**	Koundaya	0/81 (0)	0.0–6.7	7/85 (8.2)	3.4–16.2
	Mounlouga	0/52 (0)	0.0–10.3	6/58 (10.3)	3.9–21.2
	Sékhoto	-	-	11/84 (13.1)	6.7–22.2
	**Total**	**0/133 (0)**	**0.0–4.1**	**24/227 (10.6)**	**6.9–15.3**
	**Grand Total**	**1/992 (0.1)**	**0.1–0.6**	**78/1131 (6.9)**	**5.4–8.5**

* Antibody response to the *O*. *volvulus* antigen Ov16

**Fig 2:**
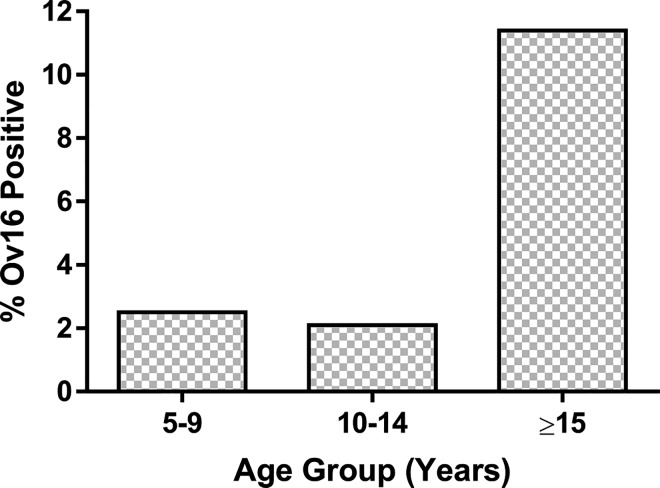
Prevalence of OV-16 Antibodies among study participants by age group

In the three river foci, the seroprevalence for Ov16 antibodies was 6.9% (n = 78, 95% CI = 5.4–8.5%). The seroprevalence among children 5–9 years old was 2.5% (n = 7, 95% CI = 1.0–5.1%) (Table 5 & Fig 2). Three of these children were from the Gambia focus and four from the Faleme focus. The participants in the age groups 15 years and older had an increased odds of having Ov16 antibodies (OR = 5.0, 95% CI = 2.3–11.1) compared to those who were 5–9 years old (Table 5).

**Table 5:** Prevalence of onchocerciasis antibody positive results by age and gender, Kédougou Region

	OV-16*		
	n/N (%)	OR	95% CI
**Age group (years)**			
5–9	7/279 (2.5)	1	
10–14	6/282 (2.1)	0.8	0.3–2.5
≥15	65/570 (11.4)	5.0	2.3–11.1
**Gender**			
Female	33/559 (5.9)	1	
Male	45/572 (7.9)	1.2	0.7–1.9

* Antibody response to the *O*. *volvulus* antigen Ov16

## Discussion

In this report, we examined whether LF testing could be added to a standard onchocerciasis epidemiological survey to provide meaningful results for both elimination programs. We found that after more than 10 years of ivermectin MDA for onchocerciasis, >1% of the participants tested were antigenemia positive for LF in one (Salemata) of the three survey districts. It is interesting to note that this district is located in the River Gambia focus where six-monthly treatment with ivermectin had occurred for more than 10 years. Ivermectin treatment coverages in these three villages ranged from 45% to 90% (Table 1). According to WHO guidelines [14], treatment for LF will be needed for at least five more years in the Salemata district. In the two other districts (Saraya and Kédougou), none of the participants tested were ICT positive, whereas during the 2003 mapping 19% of the persons tested in one village were ICT positive. Though very encouraging, the lack of antigenemia in the other two districts should be interpreted cautiously. The design of this study was not robust enough to conclude with confidence that LF elimination has been achieved in these two districts. Based on the 2011 WHO criteria, <2% antigenemia prevalence among 6–7 year olds (transmission assessment survey [TAS]) needs to be achieved to conclude that LF transmission has been interrupted [15]. On average, about 1,500 children need to be included randomly in a TAS. The design of this study did not allow to achieve these goals. A low level of Wb123 antibody response was detected among participants, which might substantiate that *W*. *bancrofti* transmission historically occurred in the three districts. Wb123 is not used routinely by LF program and is still considered as a research tool. As such, unlike ICT, there are no guidelines or standard operation procedure for Wb123 use programmatically. We found a discordance of results between the ICT tests and the antibody tests, and also a very low antibody prevalence among participants (Table 2). The performance of Wb123 in low prevalence setting is still not well understood. It is possible that the cutoff values for the multiplex were inaccurate, leading to inaccurate prevalence estimates. The ability to define robust cutoffs for serologic assays can be challenging and is often limited by the availability of well characterized panels of samples to determine appropriate cutoffs. Other explanations could be the absence of exposure to *W*. *bancrofti* or the waning of antibody level in a population treated with ivermectin for many years. Thus, though providing sufficient information in areas where positive results were found to decide if ivermectin distribution should continue to treat LF, this study could not determine if LF transmission was absent in districts where no participants were ICT positive.

Previous studies in Senegal and Mali provided the first evidence that ivermectin MDA might eliminate onchocerciasis in some foci in Africa [5, 11]. The study by Traore *et al* [11] indicated that in the River Gambia focus, where ivermectin treatment was given bi-annually, there was neither presence of microfilariae detected in the skin of the human population nor presence of infective larvae (L3) detected in the head of *Simulium* vectors three to four years after stopping ivermectin MDA. The current study detected a 0.2% prevalence of microfilariae among the participants from the River Gambia focus using skin snip microscopy. In the River Faleme focus (Fig 1), where *Traore et al* [11] found an extremely low level of microfiladermia three to five years after the last treatment, no microfiladermia was detected among the participants of this study. Likewise, no microfiladermia was detected among the participants in the River Koalakabe focus. The results found among 5–9 years old children are more concerning. Though none of the children 5–9 years old had a positive skin snip, 2.5% (7/279) were positive for Ov16 suggesting that children 5–9 years old have been infected with or exposed to *O*. *volvulus* in the three river foci. Skin snips are no longer an accepted procedure to do evaluations for stopping MDA or assessing interruption of transmission. It is considered too insensitive and as encountered in this study, the refusal rate is high and likely to be among the same persons who also refuse MDAs. The recent WHO Guidelines for stopping mass drug administration and verifying elimination of human onchocerciasis [16] recommends that Ov16 serology test to determine the presence of IgG4 antibodies to the antigen Ov16 should be used in children less than 10 years of age to demonstrate the interruption of transmission of *O*. *volvulus* in population receiving MDA against onchocerciasis for the purpose of stopping MDA. Generally, a sample size of 2000 children is needed to detect a prevalence of less than or equal to 0.1% at the upper bound of the 95% confidence interval. Only 279 children 5–9 years old were included in our study but we found a prevalence of positive Ov16 serologic test among children 5–9 years old 25 times higher than the threshold recommended by the new guidelines. Therefore, this area failed to demonstrate elimination based on the new WHO criteria. A more statistically robust study in conjunction with vector collection would allow for an exploration of the status of transmission and contribute to an assessment of the current serologic threshold for the transmission of onchocerciasis.

The current study has limitations. The study essentially employed APOC onchocerciasis-related methodology, which included selection of villages based on their proximity to riverine vector breeding sites and convenience sampling. In addition, no clear sample size was determined before the study and the expectation was to include as many eligible persons present in the villages as possible. Only half of the eligible population, based on the census conducted before the study, was included in the study. Oral reports mentioned that many villagers were working elsewhere or that students were absent from the village to attend school in larger localities, but the study did not systematically ascertain the reasons for this low level of inclusion. Of the people who consented to participate, 14% refused the skin snip examination as opposed to <1% who refused to provide capillary blood. It is possible that the people who refused the skin snip were also more likely to not participate in MDAs, which would have biased the results of this study towards participants more likely to be skin snip negative. The skin snip method is more invasive and uncomfortable than capillary blood draw, which possibly explains the poorer compliance for providing skin snip biopsies, especially in communities like the ones selected for this study where people have been subjected to repeated skin snips over the years. The use of less invasive diagnostic tools for onchocerciasis elimination programs for endpoint assessment [17] will likely increase community participation in epidemiological surveys.

LF and onchocerciasis have overlapping endemicity in many parts of Africa but LF mapping and treatment have unfortunately not happened so far in many districts that have been treated for onchocerciasis. In the nearly 1,500 districts receiving MDA for onchocerciasis in Africa, 588 districts still need to be mapped for LF and 365 districts known to be co-endemic have not started treatment for LF [6]. In some of these districts, it may not be necessary to ever start treatment with albendazole if the districts were to be shown to have already reached interruption of LF transmission. It may be more efficient to assess the two diseases at the same time, especially in areas that have undergone treatment for onchocerciasis and where stop-MDA decisions need to be taken. Recommendations exist to assess onchocerciasis [16] and LF [18] separately, but they are resource intensive and to date there is no clear consensus on how to coordinate the evaluation activities of both diseases in co-endemic areas. This survey demonstrated the feasibility of collection of samples needed for an integrated evaluation of LF and onchocerciasis. It suggested strongly that LF transmission was still present in one of the three districts evaluated despite years of ivermectin distribution for onchocerciasis but the design used could not determine with statistical confidence if the infection rate for *W*. *bancrofti* was below the transmission threshold in the districts where no participants were ICT positive. Similarly, the Ov16 prevalence among 5–9 year old children strongly suggests that onchocerciasis transmission was still present and confirms the need for more robust epidemiological studies using more sensitive diagnostic tools like the Ov16 serological test [16] before the decision to stop ivermectin-based MDA for onchocerciasis can be made safely. The Ministry of Health of Senegal decided to start MDA with albendzole and ivermectin in 2015 in the three district of the Kédougou region. This study provides another example of the complexities that encompass the stop MDA decision in onchocerciasis and LF co-endemic areas [19]. This challenge was described by Katabarwa *et al* in the Wadelai focus of Uganda [20] where ivermectin-based MDA for onchocerciasis could not be stopped due to ongoing transmission of LF. In countries where co-endemicity exists, coordination of onchocerciasis and LF elimination efforts are needed especially when MDA stopping decisions are concerned [6].

## Supporting Information

S1 Checklist STROBE Checklist (DOCX)
